# Mapping Copper and Lead Concentrations at Abandoned Mine Areas Using Element Analysis Data from ICP–AES and Portable XRF Instruments: A Comparative Study

**DOI:** 10.3390/ijerph13040384

**Published:** 2016-03-30

**Authors:** Hyeongyu Lee, Yosoon Choi, Jangwon Suh, Seung-Ho Lee

**Affiliations:** 1 Department of Energy Resources Engineering, Pukyong National University, Busan 48513, Korea; lhg6197@gmail.com; 2 Department of Energy and Mineral Engineering, The Pennsylvania State University, PA 16802, USA; jangwonsuh@hanmail.net; 3 Mine Reclamation Corporation, Wonju 26464, Korea; cortoimasa@mireco.or.kr

**Keywords:** portable X-ray fluorescence, inductively coupled plasma atomic emission spectrometry, ordinary kriging, co-kriging, soil contamination map

## Abstract

Understanding spatial variation of potentially toxic trace elements (PTEs) in soil is necessary to identify the proper measures for preventing soil contamination at both operating and abandoned mining areas. Many studies have been conducted worldwide to explore the spatial variation of PTEs and to create soil contamination maps using geostatistical methods. However, they generally depend only on inductively coupled plasma atomic emission spectrometry (ICP–AES) analysis data, therefore such studies are limited by insufficient input data owing to the disadvantages of ICP–AES analysis such as its costly operation and lengthy period required for analysis. To overcome this limitation, this study used both ICP–AES and portable X-ray fluorescence (PXRF) analysis data, with relatively low accuracy, for mapping copper and lead concentrations at a section of the Busan abandoned mine in Korea and compared the prediction performances of four different approaches: the application of ordinary kriging to ICP–AES analysis data, PXRF analysis data, both ICP–AES and transformed PXRF analysis data by considering the correlation between the ICP–AES and PXRF analysis data, and co-kriging to both the ICP–AES (primary variable) and PXRF analysis data (secondary variable). Their results were compared using an independent validation data set. The results obtained in this case study showed that the application of ordinary kriging to both ICP–AES and transformed PXRF analysis data is the most accurate approach when considers the spatial distribution of copper and lead contaminants in the soil and the estimation errors at 11 sampling points for validation. Therefore, when generating soil contamination maps for an abandoned mine, it is beneficial to use the proposed approach that incorporates the advantageous aspects of both ICP–AES and PXRF analysis data.

## 1. Introduction

Mining is a global industry that can be hazardous to public health and safety, and can cause damage to the surrounding environment, including land, soil, water, and forests [1]. Among the various environmental impacts of mining, contamination of soil is significant because mine waste produced by metal-mining activities generally contains higher content of potentially toxic trace elements (PTEs) than that in regular industrial waste. These substances can become widely dispersed throughout mining areas unless proper measures for isolation or treatment are taken [2,3]. Elevated concentrations of PTEs in soil do not only impact the soil quality, but due to their persistent nature and long biological half-lives, can accumulate in the food chain and can eventually influence human health [4]. Therefore, the type, content and spatial variation of PTEs in soil should be regularly investigated at both operating and abandoned mining areas to identify the proper measures necessary for preventing soil contamination [5,6].

Inductively coupled plasma atomic emission spectrometry (ICP–AES) is one of the precise and representative instruments to investigate the type and content of PTEs in soil [7]. Because the ICP–AES instrument provides a high degree of accuracy with regard to chemical element analysis [8], it has been widely used to investigate the soil quality in mining areas. However, the ICP–AES analysis has several disadvantages such as its costly operation and lengthy period required for analysis owing to the complex preprocessing process of soil drying, crushing, sieving, and acid digestion for alteration from a solid to liquid-phase state [9]. Therefore, the type and content of PTEs in soil can be investigated only at certain sampling points due to cost and time constraints.

To compensate for such disadvantages, an on-site analysis method employing a portable X-ray fluorescence (PXRF) instrument has been specified in the U.S. Environmental Protection Agency’s (EPA’s) method 6200 for investigating the type and content of PTEs in soil [7]. Higueras *et al.* [10] reported that the PXRF analysis is cost-effective for environmental studies. Moreover, the period required for element analysis can be reduced significantly since the PXRF analysis does not necessitate the complex preprocessing process of soil sample [11,12]. Therefore, using the PXRF instrument, the type and content of PTEs in soil can be investigated at much more sampling points compared with the ICP–AES instrument within same cost and time constraints. However, the PXRF analysis has relatively low accuracy compared to ICP–AES analysis [12]. 

Regardless of the equipment used for analysis of the type and content of PTEs in soil, it is generally difficult to understand the spatial variation of PTEs for an entire mining area because the soil quality is investigated at more or less sparse sampling points. Geostatistical methods provide a valuable tool to study the spatial variation and to generate soil contamination maps [13,14,15]. They take into account spatial autocorrelation of data to create mathematical models of spatial correlation structures commonly expressed by semi-variograms [16]. The interpolation technique of the variable at unsampled locations, known as kriging, provides the “best”, unbiased, linear estimate of a regionalized variable in an unsampled location, where 'best' is defined in a least-squares sense [15].

Many studies have been conducted worldwide to explore the spatial variation of PTEs and to generate soil contamination maps at local and regional scales using geostatistical methods [17,18,19,20,21,22]. They usually depend only on ICP–AES analysis data, therefore such studies are limited by insufficient input data owing to the disadvantages of ICP–AES analysis. Although several attempts have been made to use PXRF analysis data as input data for exploring the spatial variation of PTEs using geostatistical methods [6,9,10,12], the relatively low accuracy of PXRF analysis data still makes it difficult to generate soil contamination maps of high quality. Until recently, few studies have attempted to use both ICP–AES and PXRF analysis data to compensate for any disadvantages and to investigate the spatial variation of PTEs by generating soil contamination maps. The approach that incorporates the advantageous aspects of both ICP–AES and PXRF analysis data may be an efficient option for exploring the spatial variation of PTEs using geostatistical methods when the amount of ICP–AES analysis data is insufficient or the accuracy of PXRF analysis data is relatively low. To assess its feasibility, it is necessary to compare the approaches that use either ICP–AES or PXRF analysis data with those that use both of them for creating soil contamination maps.

Against this background, the aim of this study was to compare the prediction performances of four different approaches for mapping copper and lead concentrations at a section of the Busan abandoned mine in Korea using element analysis data from ICP–AES and PXRF instruments. The four approaches include: (1) the application of ordinary kriging to ICP–AES analysis data; (2) PXRF analysis data; (3) both ICP–AES and transformed PXRF analysis data by considering the correlation between the ICP–AES and PXRF analysis data; and (4) co-kriging to both ICP–AES (primary variable) and PXRF analysis data (secondary variable). Approaches 1 and 2 use either ICP–AES or PXRF analysis data, and 3 and 4 use both of them for generating soil contamination maps. Their results were compared using an independent validation data set. 

## 2. Materials and Methods

### 2.1. Study Area and Soil Sampling

**Figure 1:**
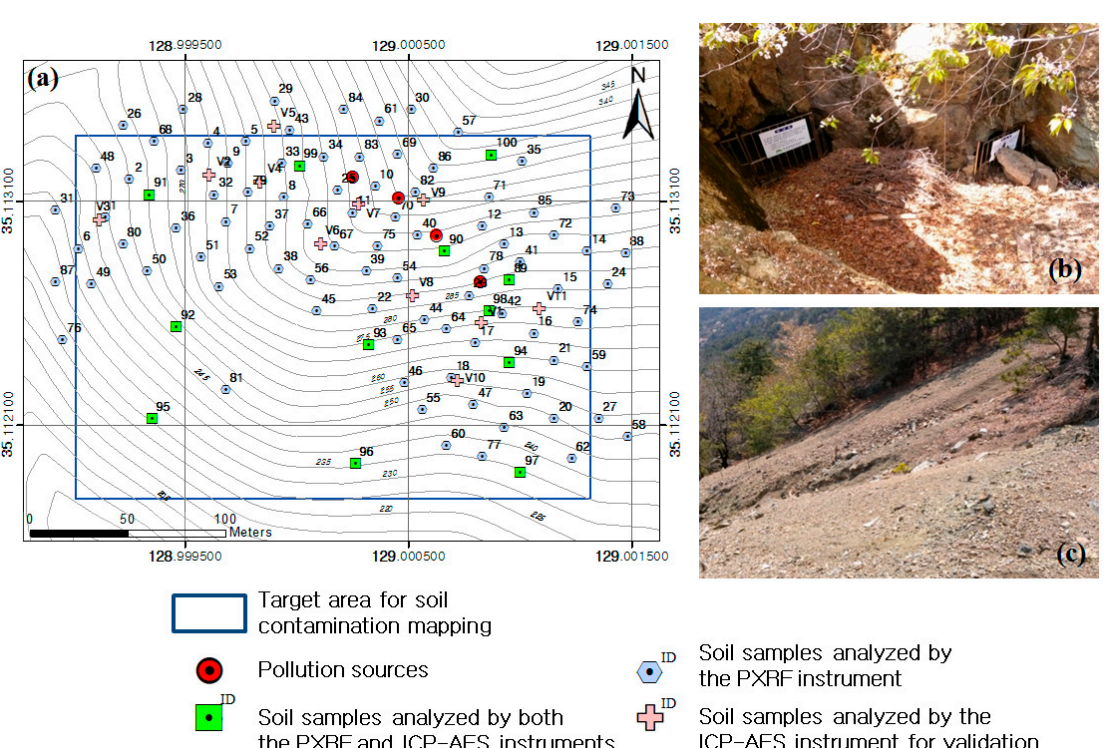
Study area: (**a**) Boundary of target mapping area (128°59ʹ56.433ʹʹ–129°0’4.726” E, 35°6’42.377”–35°6’48.218” N) and the locations of contamination sources and soil sampling for the ICP–AES and PXRF analyses. The extent of the soil sampling area is larger than that of the target mapping; (**b**,**c**) Photographs of closed pit heads and mine waste rocks on the slope, respectively.

This study selected part of the Busan mine located at Saha-gu, Busan, South Korea, as a target area (Figure 1). Currently abandoned, the Busan mine was in operation until 1986 and produced 2246 tons of iron. About 4000 m^3^ of mine waste rocks and tailings piled around the pit heads have not undergone proper environmental treatment (Figure 1); high concentrations of copper and lead were found near the waste rock pile and pit heads [23]. Furthermore, it is estimated that the soil contamination due to mine waste rocks and tailings has been dispersed downslope by surface erosion.

By using a PXRF instrument (Innov-X DELTA handheld XRF analyzer, Olympus, Japan), on-site analysis for copper and lead contents was conducted at 100 points. This PXRF instrument is equipped with an Au anode as the excitation source and a silicon drift detector, and operates at 40 kV and 0.1 mA. The sampling points and topography of the study area are shown in Figure 1. Surface soils down to 10 cm in depth were sampled by using a hand auger at the points. Each soil sample included a composite of nine subsamples taken within a 5 m × 5 m area. The soil samples were then disaggregated and sieved to <2 mm in the field. The prepared soil samples were analyzed as loose powders by PXRF, using a sample cup fitted with a 6 µm thick polyester film. The procedure for quantification was conducted by software embedded in the PXRF instrument.

It is noteworthy that the results of element analysis by the PXRF instrument can vary depending on the water content in the soil [8,9]. Tolner *et al.* [9] determined that the metallic element content detected will be about 1%–3%, 23%–30%, and 30%–39% lower when the water content is 10%, 15%, and 20%, respectively, than that in completely dried soil. Therefore, in this study, the water content in each soil sample was measured using a portable soil moisture meter (PMS-714, Lutron, Taiwan), and element analysis by the PXRF instrument was then conducted when the soil sample had a water content of less than 10%. The detection limits and measurement errors of PXRF instrument are also dependent on analysis time; longer analysis times generally provide lower detection limits and measurement errors [9]. In this study, the PXRF analysis was conducted once per sample for 2 min because the measurement error showed a tendency to decrease to less than 30% and to less than 20% when the measurements last 1 min and 2 min, respectively [9].

In addition, soil samples were collected at 23 points to analyze copper and lead contents by using an ICP–AES instrument (VISTA-PRO, Varian, CA, USA). The soil samples consisted of 12 samples collected at the points in which the PXRF analysis was previously conducted and 11 samples collected at random points for validating the prediction performances of four different approaches for mapping copper and lead concentrations (Figure 1). The preprocessing of 23 soil samples was conducted in the laboratory. After air-drying at 25 °C for five days, the soil samples were disaggregated and sieved to <2 mm and then ground to a fine powder (<2 μm). The samples were then digested into 0.1 N of HCl solution, with 10 g of soil in 50 mL of the solution, according to the Korean standard method for the chemical analysis of soils. The solutions were analyzed by using the ICP–AES instrument for two elements including copper and lead. The quantification of each element analyzed by ICP–AES was performed using a calibration curve. In addition, the pH and electrical conductivity (EC) of soil samples were measured in the laboratory.

### 2.2. Geostatistical Methods

In Geostatistics each measured value, z(x_i_), at location x_i_ (x is the location coordinates vector and i = 1,…, N indicates the sampling points) is interpreted as a particular realization of a random variable Z(x_i_). The set of dependent random variables {Z(x_i_), i = 1,…, N} constitutes a random function Z(x) [14,16]. 

The experimental variogram γ(h) is an important tool in Geostatistics that measures the variability between two data pair values [z(x_i_), z(x_i_ + h)] of the same variable z(x_i_) separated by a lag vector h. It is a function of the lag h, a vector in distance and direction, of the two data pair values, and can be calculated as follows.

(1) $$\gamma \left(h\right)=\frac{1}{2N}\sum_{i=1}^{N}{\left[z\left(x_{i})-z(x_{i}+h\right)\right]}^{2}$$

where N is the number of data pairs for a given class of distance and direction. A function, known as the theoretical variogram model, is fitted to the experimental variogram to allow one to analytically estimate the variogram for any distance h. The function should be conditionally negative definite to ensure that the kriging variances are positive [13]. The best fitting function can be selected by cross-validation which checks the compatibility between the data and the model. The variogram model can be extended to multiple variables. If we considered N regionalized variables, there are N direct and N(N + 1)/2 cross variograms required. These direct and cross variograms should share some features and cannot be considered independently [24]. Interested readers should refer to textbooks such as [13,15,25].

The most typical interpolation method used in Geostatistics is kriging [18]. Kriging is a technique in which an unknown value with a weighted linear combination of already known surrounding values is predicted.

(2) $$z^{*}=\sum_{i=1}^{N}\lambda_{i}z_{i}$$

where z* is the kriging predicted value at the prediction point, z_i_ is a data value of the vicinity, for which the location and value are already known, λ_i_ is the weight of each data, and N is the total number of data used for the kriging prediction. According to a method determining weight, several types of kriging can be classified: simple kriging, ordinary kriging, universal kriging and kriging with external drift [26].

Ordinary kriging is the most common geostatistical estimator [18]. It mainly used when data satisfy a weak secondary invariance and do not show a particular trend. When predicting an unknown value using ordinary kriging, the error variance is minimized while the kriging estimation equation is not biased. Bias is defined as a difference between the factor average of the population and the estimation equation average for predicting that population factor; when there is no difference, it is said to be unbiased. A condition for the equation not to become biased is as follows:

(3) $$b_{z^{*}}=E\left(z\right)-E\left(z^{*}\right)=0$$

where $b_{z^{*}}$ shows a bias of z*, and since all data used in kriging have an identical average value, the sum of the weights must be 1. This is shown in the following equation in order for the kriging estimation equation to be always unbiased:

(4) $$1-\sum_{i=1}^{n}\lambda_{i}=0$$

Ordinary kriging was selected as a spatial interpolation technique in this study because existing reports indicate that the soil contamination distribution of copper and lead in the Busan mine area satisfy a weak secondary invariance and do not show a particular trend [23]. 

Co-kriging is a multivariate geostatistics technique in which an unknown value is predicted by using two or more variables. Using a primary variable to be predicted and a secondary variable having a correlation with the primary variable, a weight is determined and the distribution of the primary variable is determined. A general equation of co-kriging is as follows:

(5) $$z^{*}=\sum_{i=1}^{N}\lambda_{i}z_{i}+\sum_{i=1}^{M}k_{j}u_{j}$$

where z_i_ is a primary variable, N is the number of data for the primary variable, λ_i_ is the weight of the primary variable, u_j_ is a secondary variable, M is the number of data for the secondary variable, and k_j_ is the weight of the secondary variable. When the number of primary variables is small and the number of secondary variables with relatively low accuracy is large, co-kriging is usually used [18]. A spatial correlation should exist between the two variables, and using the secondary variable can reduce the uncertainty of the predicted value for the primary variable. Thus, co-kriging is known to be suitable for geostatistical integration of two data with complementary characteristics. To apply co-kriging, there should be a variogram for each variable, and a cross variogram between the primary and secondary variables is necessary.

### 2.3. Four Different Approaches for Mapping Copper and Lead Concentrations

Four different approaches based on ordinary kriging and co-kriging were used to generate soil contamination maps for copper and lead by using the ICP–AES and PXRF analysis data (Table 1). Even if ordinary kriging and co-kriging do not require the input data to follow a normal distribution, the variogram is sensitive to strong departure from normality because a few large values can contribute to many very large squared differences. Such skewness can often be removed, and the variances stabilized by transforming the data to their corresponding natural logarithms [14]. This leads to lognormal kriging [27]. Therefore, this study transformed the element analysis data from ICP–AES and PXRF instruments to their corresponding natural logarithms if their histograms are characterized by a positively skewed distribution.

Approaches 1 and 2 were designed to generate soil contamination maps using either the ICP–AES (*n* = 12) or PXRF (*n* = 100) analysis data. The ICP–AES analysis data from 11 validation samples were not utilized herein. Because approaches 1 and 2 depended on a single data source, they are almost identical to the conventional approaches used in the previous studies for soil contamination mapping. Ordinary kriging was used as a spatial interpolation technique for approaches 1 and 2. 

Conversely, approaches 3 and 4 were designed to generate soil contamination maps by using both ICP–AES and PXRF analysis data. In approach 3, the PXRF analysis data, which has relatively low accuracy, were transformed by considering the correlation between the PXRF and ICP–AES analysis data. This study examined the correlation by using the element analysis data of 12 samples in which the PXRF and ICP–AES analyses were performed together. After the transformation, ordinary kriging was applied to the ICP–AES (*n* = 12) and transformed PXRF analysis data (*n* = 100) to generate copper and lead soil contamination maps. The ICP–AES analysis data was used in preference when both the ICP–AES and transformed PXRF analysis data were available at the same point. Ordinary kriging was also used for approach 3. In approach 4, co-kriging was applied to both ICP–AES (primary variable) and PXRF analysis data (secondary variable) to generate the soil contamination maps. Here, the PXRF analysis data, which exhibit relatively low accuracy, have a role in the reduction of the uncertainty of the estimated value from the ICP–AES analysis data if a correlation is detected.

The soil contamination maps for copper and lead generated by the four different approaches were compared by considering the spatial variations of copper and lead in the maps and the estimation errors at the 11 validation sample points (Figure 1). Because the spatial variation of PTEs in an abandoned mine area is largely affected by topography and surface erosion owing to runoff [1,28,29,30], this study used a digital elevation model (DEM) to identify the topographical and hydrological characteristics of the target area. To generate a DEM of the study area, topographical contours with 5-m intervals were extracted from 1:5000-scale topographical maps published by the National Geographic Information Institute of Korea. A triangulated irregular network (TIN) surface was then created from the topographical contours and was converted to a DEM with 5-m grid spacing using ArcGIS 10.1. Spurious depressions on the DEM were identified by ground inspection and were removed by using the Fill tool in ArcGIS. The flow directions of rainwater in the watershed area including the contamination sources were analyzed by using the DEM and the Flow Direction tool in ArcGIS.

## 3. Results and Discussion

**Figure 2:**
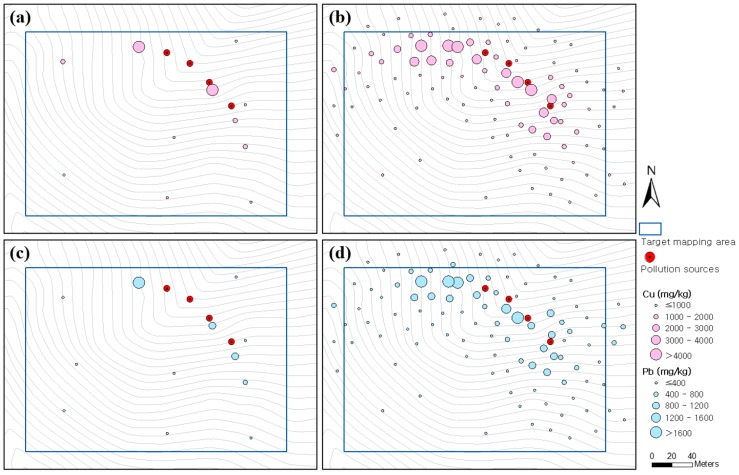
Results of element analysis: (**a**,**c**) Copper and lead contents in soil analyzed by ICP–AES, respectively; and (**b**,**d**) copper and lead contents in soil analyzed by PXRF, respectively. The ICP–AES analysis results for the 11 validation samples are not illustrated.

Figure 2 shows the spatial distribution of copper and lead contents at the 100 sampling points that were analyzed by the ICP–AES and PXRF instruments. The concentration of copper and lead were low in most of areas, although high concentrations were detected near the waste rock pile and pit heads. A statistical summary of the PXRF analysis results include copper at minimum = 21 mg/kg, maximum = 8255 mg/kg, mean = 1129.6 mg/kg, and standard deviation (std. dev.) = 1563.6 mg/kg and lead at minimum = 33 mg/kg, maximum = 2350 mg/kg, mean = 436.6 mg/kg, and std. dev. = 453.2 mg/kg. The copper and lead contents analyzed by the ICP–AES instrument for the 23 soil samples, including the 11 validation samples, are presented in Table 2. The ICP–AES analysis results showed 4437 mg/kg maximum, 17 mg/kg minimum and 1212.8 mg/kg mean for copper, and 1924 mg/kg maximum, 26 mg/kg minimum and 410.9 mg/kg mean for lead. The ICP–AES analysis data, with relatively high accuracy, tended to be lower than that of PXRF. The EC and pH for the 23 soil samples are also presented in Table 2. A statistical summary of the results include EC at minimum = 13.7 µS/cm, maximum = 65.0 µS/cm, mean = 34.63 µS/cm, and std. dev. = 13.44 µS/cm and pH at minimum = 4.41, maximum = 5.76, mean = 5.07, and std. dev. = 0.34. There were no statistically significant correlations among the copper and lead contents, EC and pH.

**Figure 3:**
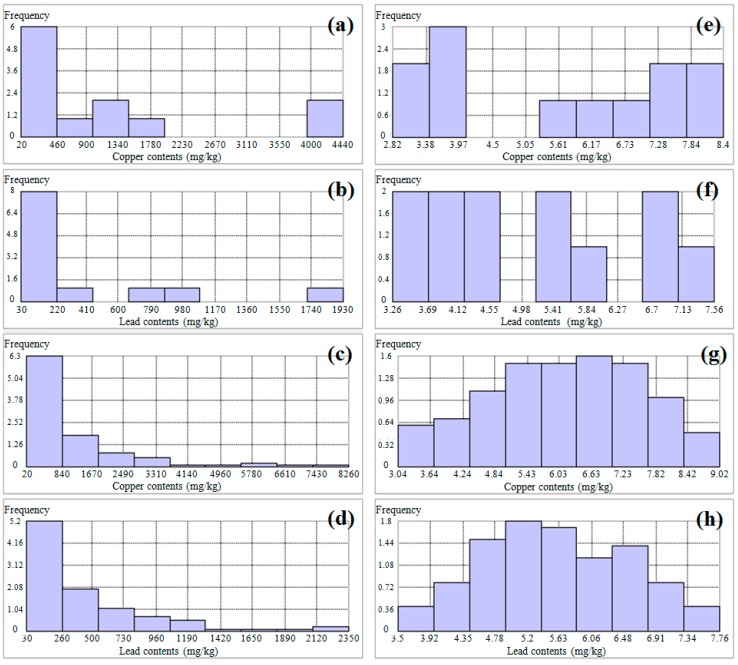
Histograms of copper and lead contents analyzed by the ICP–AES and PXRF instruments: (**a**,**b**) copper and lead by ICP–AES, respectively; (**c**,**d**) copper and lead by PXRF, respectively; (**e**,**f**) natural logarithms of copper and lead by ICP–AES, respectively; and (**g**,**h**) natural logarithms of copper and lead by PXRF, respectively.

Histograms of the copper and lead concentration are presented in Figure 3. As shown in Figure 3, the distributions of copper and lead contents measured by the ICP–AES instrument at the 12 sampling points and those measured by the PXRF instrument at the 100 sampling points were positively skewed (Figure 3a–d). After transforming the data to their corresponding natural logarithms, the PXRF analysis data for copper and lead mostly followed a normal distribution (Figure 3g,h). However, the ICP–AES analysis data for copper and lead did not followed a normal distribution due to the insufficient input data even if the natural logarithm transformations were applied to them (Figure 3e,f).

**Figure 4:**
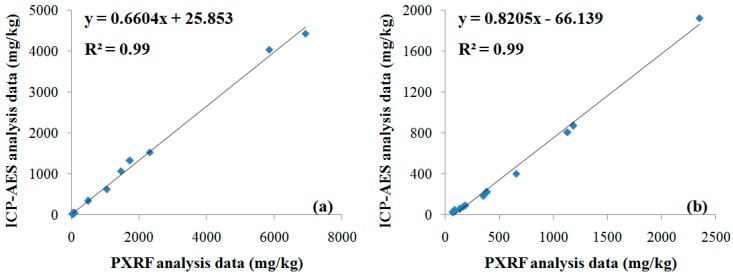
Graphs showing the correlation of ICP–AES and PXRF analysis data for: (**a**) copper; and (**b**) lead.

The correlation between the PXRF and ICP–AES analysis data was examined using the contents of copper and lead measured at the 12 sampling points in which PXRF and ICP–AES analyses were performed together. As shown in Figure 4, the value of the determination factor, R^2^, was 0.99 for copper and lead, indicating a very strong correlation. However, the PXRF analysis data, with relatively low accuracy, tended to be higher than that of ICP–AES. Therefore, the trend equations of these two data were calculated as shown in the figure and used in approach 3 to transform the overestimated PXRF analysis data (*n* = 100) into approximate values close to those determined by ICP–AES analysis.

**Figure 5:**
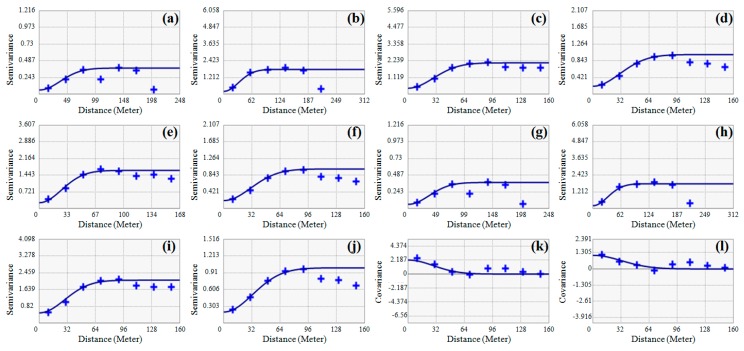
Results of variogram modeling: (**a**,**b**) copper and lead for approach 1, respectively; (**c**,**d**) copper and lead for approach 2, respectively; (**e**,**f**) copper and lead for approach 3, respectively; (**g**,**h**) copper and lead for approach 4 (primary), respectively; (**i**,**j**) copper and lead for approach 4 (secondary), respectively; and (**k**,**l**) copper and lead for approach 4 (cross variogram), respectively.

Figure 5 and Table 3 present the results of variogram modeling and cross-validation test, which is required to perform spatial interpolation by using ordinary kriging and co-kriging. A Gaussian model was selected at a fitting function for theoretical variogram model because of its general applicability when the correlation and continuity of data were strong in a small separation distance. Because a clear anisotropy appeared with the PXRF analysis data, a geometric anisotropy model was used in approaches 2 and 3. The goodness of fit was checked by cross-validation. The results were satisfactory for the PXRF (approach 2) and transformed PXRF (approach 3) analysis data, however unsatisfactory for the ICP–AES analysis data (approaches 1 and 4) due to the insufficient amount of input data. The variogram models for copper and lead obtained from the PXRF analysis data at the 12 sampling points (ID: 89–100) were almost identical to Figure 5a,b, respectively, because the correlation between the PXRF and ICP–AES analysis data was strong.

**Figure 6:**
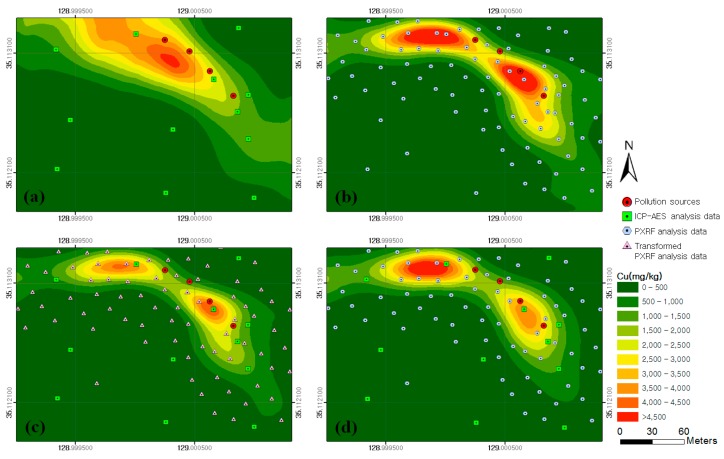
Soil contamination maps for copper generated by approaches: (**a**) 1; (**b**) 2; (**c**) 3; and (**d**) 4.

According to approaches 1–4, four types of soil contamination maps of the target mapping area were generated for copper (Figure 6). The result from approach 1 showed a northwest–southeast contamination pattern, which was estimated to be concentrated in an area of more than 1000 mg/kg in broad distribution regardless of the contamination source locations (Figure 6a). Because approach 1 depended on a small amount of ICP–AES analysis data (*n* = 12), the spreading pattern of copper concentration was not well illustrated in Figure 6a when considers the contamination sources. The result from approach 2 showed a spreading pattern of copper contamination in the western and southern directions, respectively, from the contamination sources (Figure 6b). In addition, a highly concentrated contamination area of more than 3000 mg/kg was estimated to be distributed around the contamination sources. Although the spreading pattern of copper contamination was well illustrated in Figure 6b compared with that in Figure 6a, the copper content in Figure 6b could be overestimated because the PXRF analysis data tended to be higher than the ICP–AES analysis data, as shown in Figure 4a.

Figure 6c shows the result from approach 3 in which both the ICP–AES and transformed PXRF analysis data were used. The spreading pattern of copper concentration was shown to be similar to that of Figure 6b. However, the width of the highly concentrated contamination area of more than 3000 mg/kg was estimated to be smaller than that in Figure 6b. This result occurred because approach 3 used the trend equation in Figure 4a to transform the overestimated PXRF analysis data into values close to that of ICP–AES analysis. Figure 6d illustrates the result from approach 4 in which co-kriging was applied. A spreading pattern of copper concentration similar to that shown in Figure 6c was observed. However, the width of the highly concentrated contamination area of more than 3000 mg/kg was estimated to be larger than that in Figure 6c.

**Figure 7:**
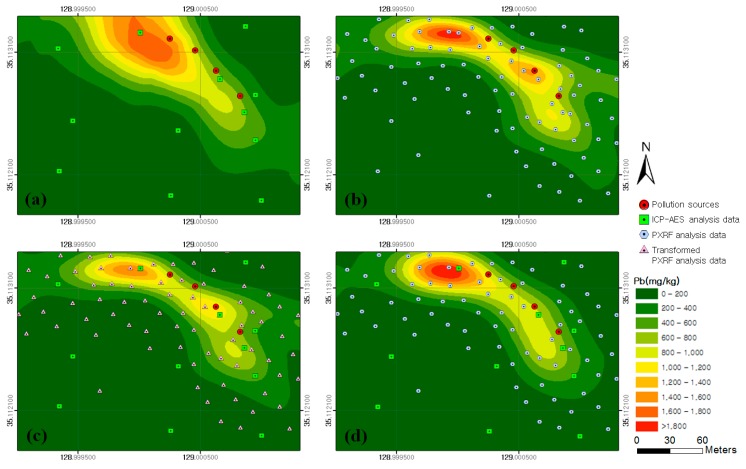
Soil contamination maps for lead generated by approaches: (**a**) 1; (**b**) 2; (**c**) 3; and (**d**) 4.

Figure 7 illustrates the soil contamination maps for lead generated by approaches 1–4. The overall spreading patterns for lead concentration shown in the four maps were similar to those of copper. In Figure 7a, the spreading pattern of lead concentration around the contamination sources was not well illustrated owing to insufficient input data. Although Figure 7b showed an improved spreading pattern of lead concentration from the contamination sources to the west and south compared with that in Figure 7a, the lead content could be overestimated owing to the limitation of PXRF data analysis. Figure 7c showed a spreading pattern of lead concentration similar to that of Figure 7b; however, a highly concentrated contamination area of more than 1400 mg/kg was not identified because the transformed PXRF analysis data was utilized in approach 3. In Figure 7d, the spreading pattern of lead concentration was similar to that shown in Figure 7c, however the width of the highly concentrated contamination area of more than 1400 mg/kg was observed.

**Figure 8:**
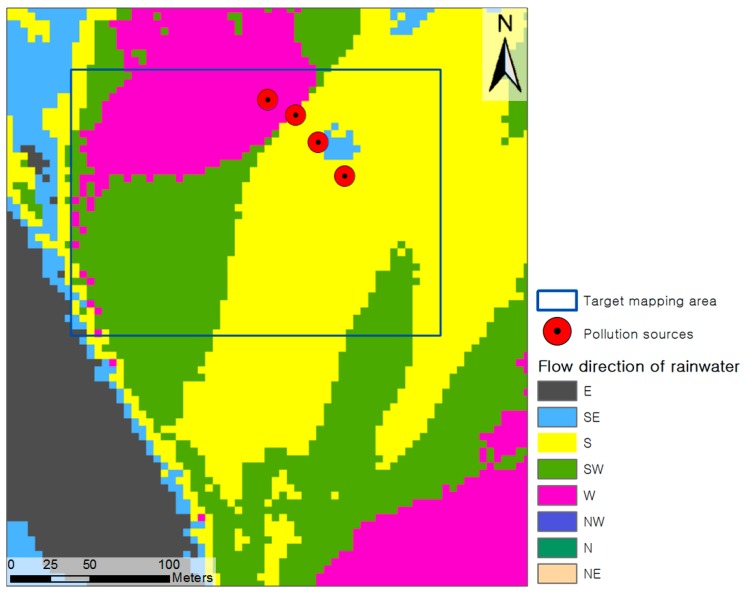
Flow direction of rainwater in the study area.

Figure 8 shows the flow direction of rainwater in the study area analyzed by using the DEM and the Flow Direction tool in ArcGIS. On the basis of the results, it was estimated that the copper and lead contaminants spread downslope from the contamination sources to the south and west. When the analytical results for the flow direction of rainwater were compared with the copper and lead soil contamination maps generated by the four approaches, the spreading patterns of contamination were shown to be similar to the results from approaches 2–4.

**Figure 9:**
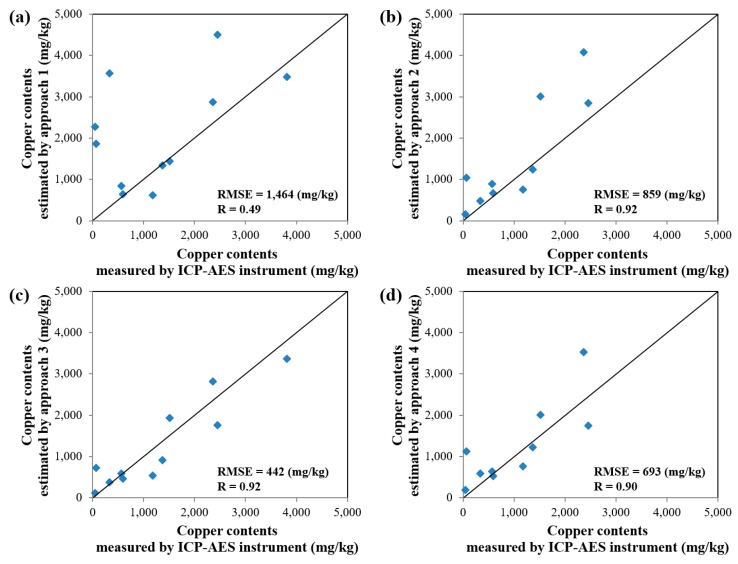
Plot showing the correlation of ICP–AES analysis data and estimated values of copper content in soil contamination maps generated by approaches: (**a**) 1; (**b**) 2; (**c**) 3; and (**d**) 4.

**Figure 10:**
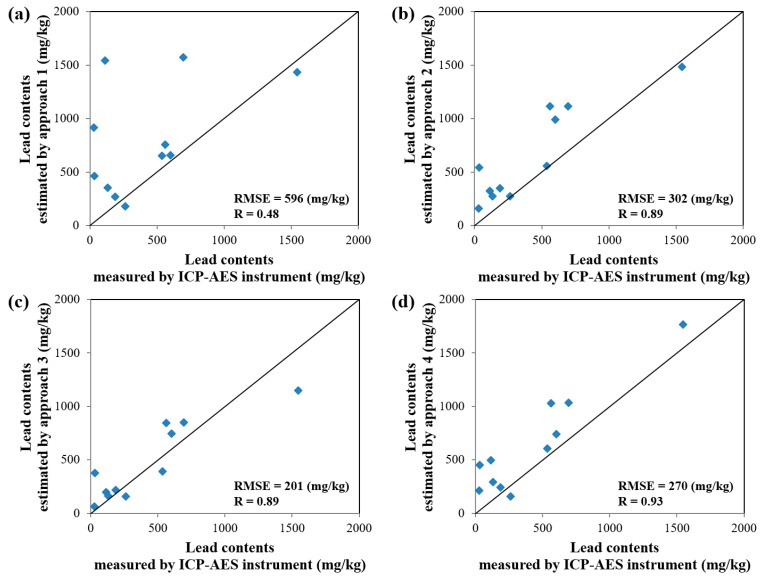
Plot showing the correlation of ICP–AES analysis data and estimated values of lead content in soil contamination maps generated by approaches: (**a**) 1; (**b**) 2; (**c**) 3; and (**d**) 4.

The estimated values of copper and lead contents in the soil contamination maps generated by the four approaches were compared with the ICP–AES analysis data for the 11 validation samples (Figure 1 and Table 2). The results of comparisons for copper and lead are illustrated in Figure 9 and Figure 10, respectively. An examination of the graphs showed that the Pearson Product–Moment Correlation Coefficient (R) values for copper and lead had strong correlations in the results from approaches 2, 3 and 4. In addition, the root-mean-square error (RMSE) was smallest in the result from approach 3 for both copper and lead, followed in order by approaches 4, 2, and 1. Therefore, we could know that the spatial variations of copper and lead contents estimated by approach 3 were more reliable than those estimated by other approaches. 

Through comparative analysis, approach 3 was determined to be accurate for the generation of soil contamination maps for copper and lead that can precisely identify the spatial variation of soil contaminants and can provide the smallest estimation error on average by incorporating the advantageous aspects of both ICP–AES and PXRF analysis data. Because co-kriging is a suitable spatial interpolation technique for the integration of multiple-source data with complementary characteristics, approach 4 was expected to best complement the advantages of the PXRF and ICP–AES analysis data. However, the errors of estimated values at the 11 validation sampling points were relatively larger than those from approach 3. This result occurred because the amount of ICP–AES analysis data (*n* = 12) for the primary variable was too small to overcome the limitation despite using the PXRF analysis data for the secondary variable. Therefore, it is necessary to examine the applicability of approach 4 if the amount of ICP–AES analysis data is insufficient.

Because the distributions of copper and lead contents measured by the ICP–AES instrument at the 12 sampling points did not followed a normal distribution after natural logarithm transformations and their results of variogram modeling were relatively poor, the geostatisitcal methods (ordinary kriging and co-kriging) may be misused in approaches 1 and 4. However, this situation can often be occurred if we depend only on ICP–AES analysis data for exploring the spatial variation of PTEs using geostatisitcal methods. Therefore, the approach that incorporates the advantageous aspects of both ICP–AES and PXRF analysis data is recommended when the amount of ICP–AES analysis data is insufficient.

## 4. Conclusions

In this study, we compared the prediction performances of four different approaches for mapping copper and lead concentrations using element analysis data from ICP–AES and PXRF instruments. Through a comparative analysis that considered the spatial variations of copper and lead concentrations in the soil and the estimation errors at the points of 11 validation samples, the proposed approach 3, which applies ordinary kriging to both ICP–AES and transformed PXRF analysis data by considering the correlation between the ICP–AES and PXRF analysis data, was determined to be the most accurate method in generating soil contamination maps because it can incorporate the advantageous aspects of both data types.

To identify the spatial variation of PTEs in soil, it is important to generate an accurate soil contamination map using available data and an appropriate spatial interpolation method. However, numerous previous studies that use either the ICP–AES or the PXRF analysis data for generating soil contamination maps were limited by insufficient input data or the low accuracy of element analysis, respectively. From a realistic aspect, it is difficult to secure a large quantity of ICP–AES analysis data to generate a soil contamination map owing to time and cost constraints. Moreover, although the PXRF instrument can provide the additional input data needed for performing a spatial interpolation, its relatively low accuracy makes it difficult to ensure the quality of soil contamination maps. Therefore, it is beneficial to use the proposed approach 3, which utilizes both the ICP–AES and PXRF analysis data to compensate for any disadvantages when generating soil contamination maps for an abandoned mine. 

It is clear that ICP–AES analysis is the most accurate method for the assessment of soil contamination related to PTEs. However, this does not detract from the value of the proposed approach described in the paper because soil contamination mapping requires additional resources. The proposed mapping approach can act as a valuable filter for the identification of critical areas in which a more detailed investigation by using the ICP–AES instrument is necessary for the establishment of preventative measures against the spread of soil contaminants in a given area.

## Figures and Tables

**Table 1:** Four different approaches designed in this study to generate soil contamination maps for copper and lead by using the inductively coupled plasma atomic emission spectrometry (ICP–AES) and portable X-ray fluorescence (PXRF) analysis data.

ID	Input Data	Geostatistical Method
1	ICP–AES analysis data	Ordinary kriging
2	PXRF analysis data	Ordinary kriging
3	ICP–AES and PXRF analysis data transformed by considering the correlation between them	Ordinary kriging
4	ICP–AES (primary) and PXRF (secondary) analysis data	Co-kriging

**Table 2:** Results of element analysis by using the ICP–AES instrument for 23 soil samples. Electrical conductivity (EC) and pH of the soil samples are also provided. The sample locations are shown in Figure 1.

ID	Cu (mg/kg)	Pb (mg/kg)	EC (µS·cm)	pH	Remark
89	636	188	44.0	5.76	12 samples in which the PXRF and ICP–AES analyses were performed together
90	4437	811	17.8	5.69	
91	1080	223	26.3	5.04	
92	17	26	27.1	4.72	
93	57	81	32.4	4.56	
94	1338	402	36.4	5.01	
95	33	59	51.3	4.80	
96	29	46	30.1	5.57	
97	344	94	27.6	4.68	
98	1535	872	13.7	5.32	
99	4041	1924	22.4	4.93	
100	48	29	48.3	5.09	
V1	1517	602	29.7	4.95	11 samples collected at random points for validating the soil contamination maps
V2	2364	562	26.7	5.01	
V3	1176	262	35.4	5.20	
V4	3814	1545	30.3	4.95	
V5	334	114	38.2	4.89	
V6	48	29	59.6	4.88	
V7	2454	695	65.0	4.41	
V8	1368	536	31.7	5.10	
V9	69	32	14.5	5.47	
V10	589	132	37.1	5.21	
V11	567	187	50.9	5.38	

**Table 3:** Variogram modeling parameters used for the generation of soil contamination maps and results of cross-validation test.

ID of Approach	Element	Model	Type	Nugget	Sill	Range (Major/Minor)	Major Direction ^1^	Cross-Validation	
								ME ^2^	RMSE ^3^
1	Cu	Gaussian model	Isotropic model	0.6	3.2	90	-	475	2392
	Pb			0.3	2.2	80		88	611
2	Cu		Geometric anisotropic model	0.4	1.7	70/35	115	232	588
	Pb			0.2	0.8	75/40	115	35	170
3	Cu		Geometric anisotropic model	0.25	1.4	65/40	115	86	330
	Pb			0.2	0.8	75/40	115	29	140
4 (primary)	Cu		Isotropic model	0.6	3.2	90	-	572	1044
	Pb			0.3	2.2	80		56	248
4 (secondary)	Cu			0.5	1.6	90		-	-
	Pb			0.5	0.7	80		-	-
4 (cross variogram)	Cu			N/A	2.2	90		-	-
	Pb			N/A	1.0	80		-	-

^1^ Parameters required for geometric anisotropic models; ^2^ Mean error (mg/kg); ^3^ Root-mean-square error (mg/kg).

## Abbreviations

The following abbreviations are used in this manuscript:

PTEs	Potentially toxic trace elements
ICP–AES	Inductively coupled plasma atomic emission spectrometry
PXRF	Portable X-ray fluorescence
DEM	Digital elevation model
RMSE	Root-mean-square error
